# Granulocyte-Macrophage Colony-Stimulating Factor in Combination With Chemoradiation for Recurrent or Metastatic Cervical Cancer

**DOI:** 10.7759/cureus.54573

**Published:** 2024-02-20

**Authors:** Na Zhang, Ming-Fang Guo

**Affiliations:** 1 Department of Gynecologic Oncology, Chongqing Key Laboratory of Translational Research for Cancer Metastasis and Individualized Treatment, Chongqing University Cancer Hospital & Chongqing Cancer Institute & Chongqing Cancer Hospital, Chongqing, CHN

**Keywords:** platinum-based chemotherapy, radiotherapy (rt), myeloid dendritic cells, uterine cervical cancer, granulocyte-macrophage colony-stimulating factor

## Abstract

Recurrent or metastatic cervical cancer carries a bleak prognosis and presents a formidable challenge in terms of treatment. Granulocyte-macrophage colony-stimulating factor (GM-CSF) increases the body's immune response by enhancing antigen presentation, which has been rarely reported in recurrent or metastatic cervical cancer. A 44-year-old woman presented to the hospital with vaginal bleeding four years after radical hysterectomy for stage IB2 squamous cell carcinoma (SCC) of the cervix (grade II-III). Gynecological examination and imaging revealed a vaginal mass, and the biopsy confirmed the recurrence of grade III SCC. The patient was treated with chemoradiation (CRT) combined with immunoadjuvant GM-CSF and achieved complete remission and a progression-free survival of two years.

## Introduction

The impact of the tumor immune microenvironment on treatment is complex. Cytokines modulate the tumor immune microenvironment to convert cold tumors into hot tumors [1]. Granulocyte-macrophage colony-stimulating factor (GM-CSF) promotes the formation of mature dendritic cells (DCs) and induces a specific immune response in the body. GM-CSF induces M1-type macrophage production and exerts tumor-suppressive effects. GM-CSF can enhance the anti-cancer effect of T cells by modulating the innate and adaptive immune response [2]. The efficacy of GM-CSF in the treatment of recurrent or metastatic cervical cancer is uncertain. The optimal therapeutic dose of GM-CSF and the optimal combination therapy regimen still require investigation. Here, we report a patient treated with GM-CSF combined with chemoradiation (CRT) for recurrent cervical cancer.

## Case presentation

A 44-year-old otherwise healthy married and childbearing patient with no significant family history presented with vaginal bleeding for seven days, four years after radical surgery for squamous cell carcinoma (SCC) of the cervix. In April 2017, the patient presented with vaginal bleeding and was diagnosed with stage IB2 SCC of the cervix (grade II-III). Human papillomavirus (HPV) type 16 was positive. Laparoscopic radical hysterectomy, bilateral salpingo-oophorectomy, and pelvic lymph node dissection were performed on May 2, 2017. Postoperative pathology showed intermediate-low differentiated SCC of the cervix, with no carcinoma involvement at the vaginal wall margins and no lymphovascular space invasion, and HPV 16 was positive. No adjuvant treatment was performed, and no recurrence or metastasis was found on regular follow-up. The patient revisited the hospital in March 2021 due to vaginal bleeding.

Her BMI was 26.22 kg/m^2^. Gynecological examination revealed a fixed and painless mass of approximately 2.0 cm x 3.0 cm at the vaginal stump, which reached the middle of the anterior vaginal wall, with no other positive signs.

The results of tumor markers showed cancer antigen (CA) 199 of 27.21 U/mL (range, 0-34.0), SCC antigen (SCC) of 0.50 ng/mL (range, 0-1.50), and cytokeratin 19 fragment 2.05 ng/mL (range, 0-2.08). The HPV test result was high-risk type 16 positive (7.85E+06 copies/104 epithelia). Routine blood test results were as follows: white blood cell count 3.64 × 109/L (range, 3.5-9.5), platelet count 171 × 109/L (range, 125.0-350.0), and hemoglobin 117 g/L (range, 115-150). The neutrophil-to-lymphocyte ratio (NLR) was <4. Total T lymphocyte, CD8 lymphocyte, CD4 lymphocyte, natural killer (NK) cell, and B lymphocyte counts were all within the normal range. Liver and kidney function and coagulation test results were normal.

**Figure 1:**
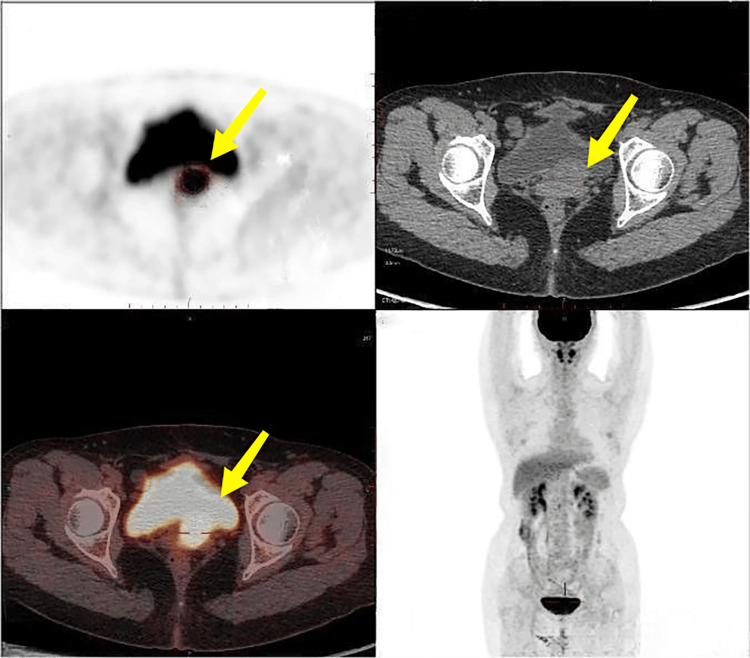
Positron emission tomography/computed tomography (PET/CT) findings Positron emission tomography/computed tomography showed a soft tissue density mass at the vaginal stump measuring approximately 3.3 cm × 3.1 cm with increased metabolism (yellow arrow).

**Figure 2:**
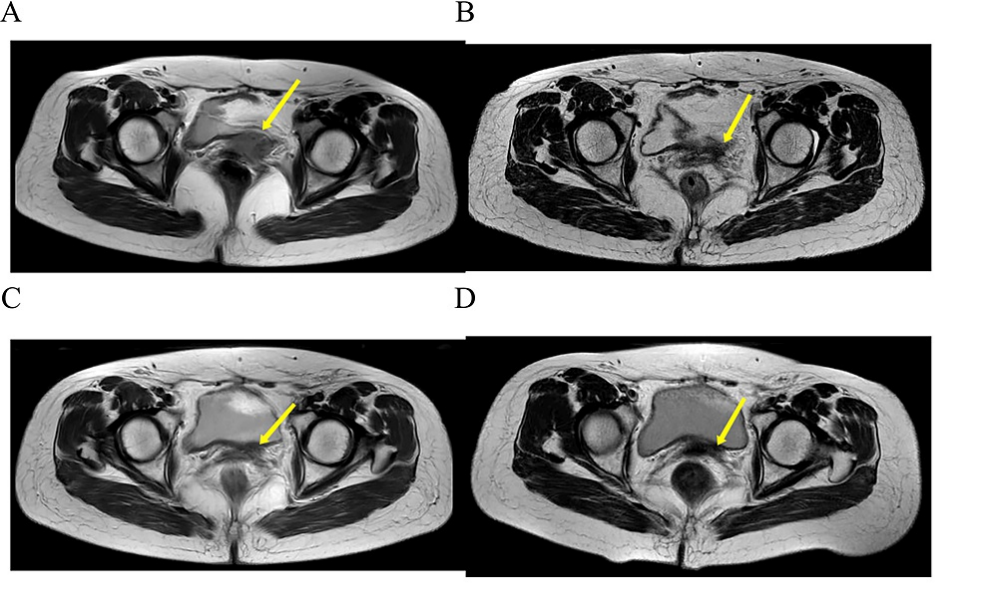
Enhanced magnetic resonance imaging of the pelvis A: Pre-treatment pelvic magnetic resonance imaging (MRI) shows a mass at the vaginal stump measuring approximately 3.0 x 2.2 cm (yellow arrow). B: Repeat pelvic MRI at three months after the end of treatment showed slight thickening of the vaginal stump. C: Repeat pelvic MRI at six months after the end of treatment showed slight thickening of the vaginal stump. D: Repeat pelvic MRI one year after the end of treatment showed slight thickening of the vaginal stump.

Positron emission tomography/computed tomography (PET/CT) showed a soft tissue density mass at the vaginal stump measuring approximately 3.3 x 3.1 cm with increased metabolism and a maximum standardized uptake value of 12.8 (Figure 1). Pelvic magnetic resonance imaging (MRI) showed a mass at the vaginal stump, measuring approximately 3.0 cm x 2.2 cm, with significant inhomogeneous enhancement on the enhanced scan (Figure 2A). Cystoscopy revealed a markedly elevated mass indentation of the left triangle and left ureteral opening with smooth surface mucosa. Extravesical lesions compressing the bladder were considered. The pathological biopsy results showed that the vaginal stump mass was grade III SCC.

Based on the patient's gynecological examination, pathological examination, imaging data, and her symptoms, she was diagnosed with recurrent cervical SCC after surgery.

After a multidisciplinary consultation including the departments of oncology, imaging, pathology, and radiotherapy, gynecological oncology was carried out to formulate comprehensive treatment plans. Informed consent was obtained from the patient for treatment. Chemotherapy with liposomal paclitaxel and cisplatin was administered one week after the start of radiotherapy, and subcutaneous GM-CSF injection was given two weeks after the start of radiotherapy. The patient underwent fixed-field intensity-modulated radiotherapy and daily image guidance from March 25, 2021 to May 27, 2021. She was irradiated with 6MV-X to the following areas: vaginal stump mass, total vagina, paravagina, and the pelvic lymphatic drainage area (including the closed foramen, presacral, internal iliac, external iliac, and common iliac). The radiotherapy dose of the planned target volume was 45 Gy/25F (1.8 Gy/F), once a day, Monday to Friday each week, for a total of 25 fractions. The patient underwent four intracavitary brachytherapy sessions from June 15, 2021 to July 20, 2021, on the vaginal plugs and total vagina, with a reference point of 0.5 cm submucosa and a single dose of 6 Gy. The cumulative radiotherapy dose to the base of the vaginal stump mass was 77 Gy. Six cycles of intravenous chemotherapy (liposomal paclitaxel 240 mg d1 + cisplatin 85 mg d2) were administered from April 1, 2021 to August 9, 2021. The patient received subcutaneous GM-CSF 200 μg daily for 14 days starting on April 8, 2021 and May 6, 2021.

**Figure 3:**
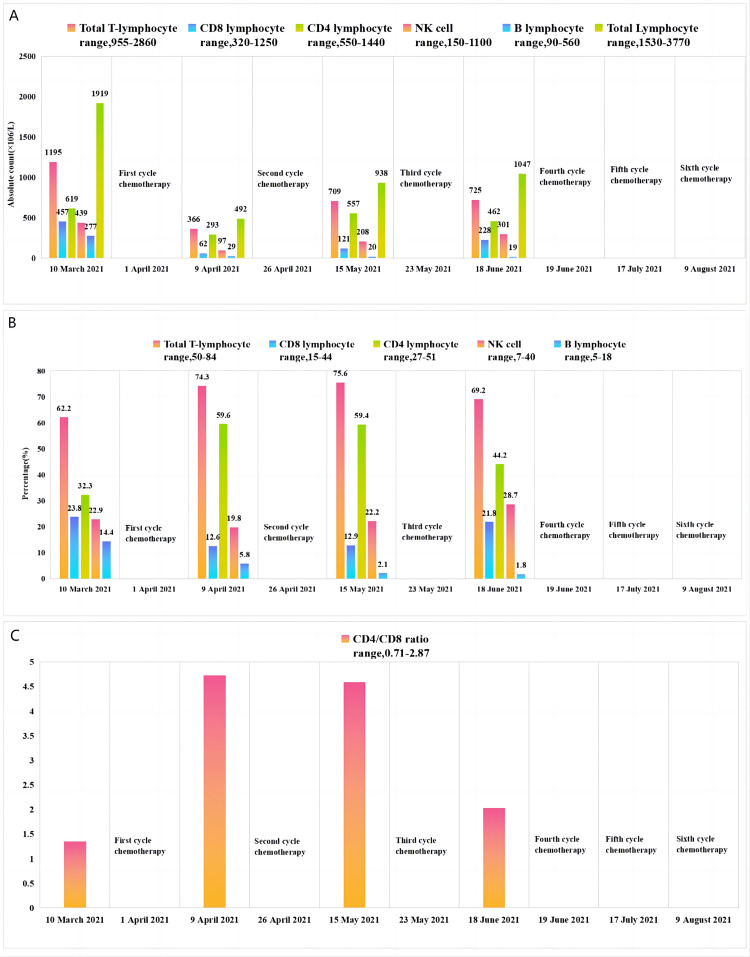
Immune cell changes before, during, and after the granulocyte-macrophage colony-stimulating factor treatment A: Change in absolute peripheral blood immune cell values from March 10, 2021 to June 18, 2021. A significant decrease in CD4+ T cells, CD8+ T cells, and NK cells in the patient was observed during granulocyte-macrophage colony-stimulating factor (GM-CSF) combined with chemoradiotherapy treatment. B: Change in the percentage of immune cells. C: Change in the CD4/CD8 ratio. An increase in the CD4/CD8 ratio was observed during treatment.

**Figure 4:**
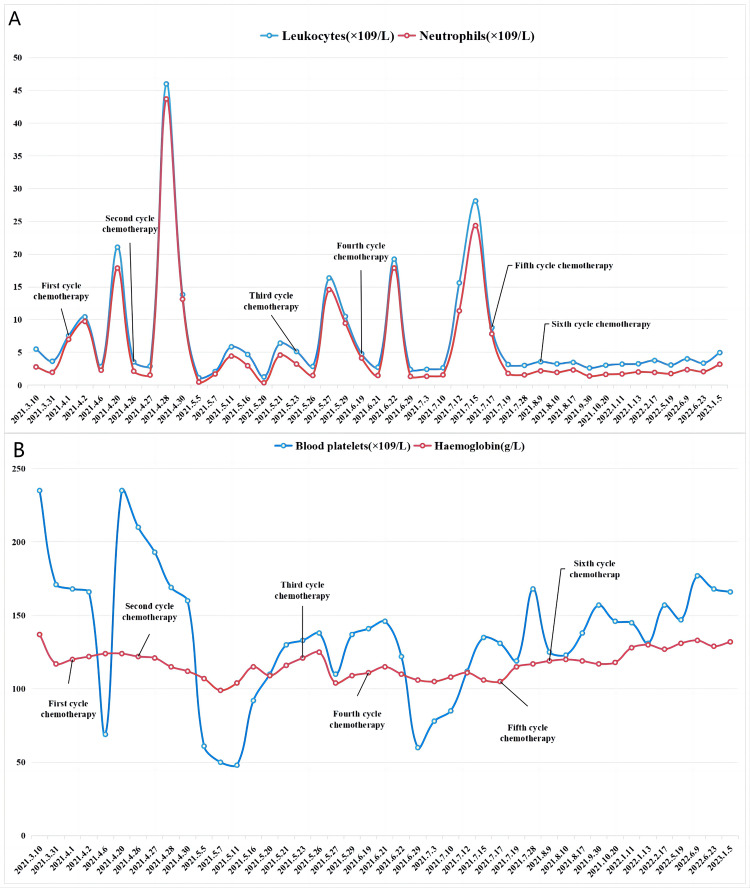
Changes in the patient's blood count. A: Changes in absolute leukocyte and neutrophil values in the patient. Both were elevated during the GM-CSF treatment. B: Changes in the patient's platelets and hemoglobin. Only mild anemia was observed during treatment, with grade II thrombocytopenia.

A repeat pelvic MRI three months after the end of treatment showed a slight thickening of the vaginal stump. The mass in the vaginal stump shown in the pre-treatment images was not seen at this time. Efficacy evaluation was complete remission. There was no evidence of recurrence at subsequent regular follow-up reviews (Figure 2B-2D). Immune cell changes before, during, and after the GM-CSF treatment are shown in Figure 3A-3C. SCC, cytokeratin 19 fragment, and HPV tests were all negative. The changes in the patient's blood cell counts are shown in Figure 4A-4B. Fever is the main adverse effect of GM-CSF. Five days after the GM-CSF injection, the patient developed a fever (39.0°C) that did not last more than 24 hours. Other causes of fever were ruled out.

## Discussion

Treatment of recurrent or metastatic cervical cancer is a clinical challenge, and the prognosis of recurrent or metastatic cervical cancer patients is poor, with a five-year overall survival rate of approximately 17% [3]. Treatment options for this group of patients are very limited. Surgery is a treatment option for patients with central recurrence, and total pelvic resection gives a five-year survival rate of about 50% for this type of recurrent cervical cancer, but it is damaging and has a significant impact on the patient's quality of life [4].

Key to the activation of the immune response by radiotherapy includes immunostimulatory cytokines, maturation of DCs, and recruitment and stimulation of T lymphocytes [5]. After radiation treatment of the tumor, antigens are continuously released and DCs are activated. APCs capture the antigen and present it to T lymphocytes to produce lymphokines, which act on tumor cells at the primary site to produce an antigen-specific immune response [6].

GM-CSF is secreted by a variety of immune and stromal cells, which stimulates the production of granulocytes and monocytes by hematopoietic stem cells via JAK2 and STAT5 to alleviate neutropenia and thrombocytopenia in post-chemotherapy patients [7]. GM-CSF has the ability to regulate the blood system and the body's immune system. GM-CSF promotes the differentiation and proliferation of DCs and M1 macrophages, promotes the upregulation of the expression of MHC class II molecules on the surface of APC cells, and enhances the activity of immune cells [8]. GM-CSF is the key link between DCs and T cells. Thus, GM-CSF indirectly activates T-cell proliferation and anti-tumor immune responses [9]. Radiotherapy combined with GM-CSF may be useful in the treatment of primary lesions and in eliminating occult microscopic lesions, reducing recurrence of metastases, and improving symptoms.

GM-CSF is currently being studied as an immune adjuvant in different types of cancer [10]. GM-CSF monotherapy or combination therapy has been studied clinically. However, the results and clinical benefits have shown inconsistencies. GM-CSF has a dual role in tumor progression and anti-tumor response. The application of GM-CSF enhances the migration of APCs and cytotoxic T lymphocytes to tumor lesions. GM-CSF is pro-immune at low doses, and high doses of GM-CSF may increase forkhead box P3+ regulatory T cells (Foxp3+ Tregs) and myeloid-derived suppressor cell (MDSC) production, thereby inducing the immune-suppressing effect.

GM-CSF was administered to patients with progressive malignant melanoma. The number and function of mature DCs were significantly increased in patients after treatment and were strongly associated with delayed tumor regression and recurrence. GM-CSF has the ability to inhibit the proliferation of cancer cells [11]. A subcutaneous dose of 125 mg/m^2^ for 14 days prolongs survival in patients with surgically resected stage III/IV melanoma by two years compared to patients not treated with GM-CSF [12]. Low-dose GM-CSF for breast cancer with a history of chemotherapy failure and recurrent ovarian and metastatic endometrial cancers resulted in 5% complete remission (CR) and 31.5% partial remission [13]. 

However, another study revealed a different clinical outcome [14]. Chemotherapy combined with GM-CSF for locoregional and metastatic gastric adenocarcinoma did not yield positive results in terms of improved treatment efficacy and prolonged survival in patients. Only when absolute granulocyte counts were below 500 cells/mL, patients were treated with GM-CSF (250 mg/m^2^/day for 14 consecutive days, starting on day 4 of chemotherapy). There may be a relationship between the timing of GM-CSF addition and less-than-expected treatment outcomes.

A phase III clinical study of limited-stage small-cell lung cancer showed a lower median overall survival in the GM-CSF combined with the chemoradiotherapy group, although the results were not statistically significant [15]. Patients in the GM-CSF combination therapy group experienced more non-hematological toxic reactions. The overall outcome of the GM-CSF combination therapy group was poor, based on the 2D radiotherapy technique and outdated chemotherapy regimen used in the trial. The results of the trial may be used as a guide to modern concurrent CRT protocols and techniques.

The latest study showed that GM-CSF combined with chemoradiotherapy can induce a systemic immune response in metastatic solid tumors, induce abscopal responses, and improve the overall survival of patients [16]. Studies of GM-CSF in combination with immune checkpoint inhibitors have shown that it is beneficial for improving overall survival and patient tolerability. Combined treatment strategies are safe and effective. In the future, combination therapy with GM-CSF as an immune adjuvant will be increasingly used in clinical practice.

The NLR can be used as a prognostic marker for certain cancer patients treated with GM-CSF [17]. CD8+ T cells and CD4+ T cells have an important role in the anti-tumor response. Infiltration of CD8+ T cells and CD4+ T cells around the tumor is associated with a better prognosis [18]. The CD4+/CD8+ ratio was significantly correlated with the efficacy of GM-CSF treatment. High CD3+ T cell, CD3+ CD4+ T cell, CD3+ T cell, CD3+ CD8+ T cell, and NK cell levels may be associated with better treatment outcomes [19].

In this report, we used GM-CSF in combination with CRT to treat a patient with recurrent cervical cancer. The NLR was less than 4 when tested before treatment. The final treatment outcome was CR, and the efficacy was maintained for 29 months. Radiation therapy induces GM-CSF expression and secretion in normal fibroblasts and tumor cells [20]. GM-CSF is often used in combination with high-dose radiotherapy. Radiotherapy for cervical cancer requires external radiotherapy combined with brachytherapy, which is a treatment modality using conventional radiotherapy doses with high-dose radiotherapy. By monitoring changes in immune cells in peripheral blood before and after treatment, we found a significant decrease in CD4+ T cells, CD8+ T cells, and NK cells in the peripheral blood of the patient during the treatment of GM-CSF combined with chemoradiotherapy and an increase in the CD4/CD8 ratio during treatment. These changes showed a relationship with the good prognosis in this patient. During the GM-CSF treatment, the patient's absolute leukocyte and neutrophil values were elevated, and both were less than 25×10９/Ｌ. Absolute leukocyte and neutrophil counts were up to 45×10９/Ｌdue to the use of short-acting granulocyte colony-stimulating factor, which does not produce immune effects. The patient only developed mild anemia during treatment and grade II thrombocytopenia. Fever is the main adverse effect of GM-CSF.

## Conclusions

GM-CSF in combination with pelvic radiotherapy and systemic chemotherapy may be a treatment option for patients with local recurrence of cervical cancer. This combined therapy not only prevented the damage caused by surgery but also improved the local control rate compared to CRT alone. The benefits and safety of CRT and GM-CSF combined with immune checkpoint inhibitor therapy in recurrent cervical cancer should be further explored in the future.
